# Purchases from family agriculture for school feeding in Brazilian capitals

**DOI:** 10.11606/s1518-8787.2020054001963

**Published:** 2020-07-20

**Authors:** Patricia Camacho Dias, Isis Ribeiro de Oliveira Barbosa, Roseane Moreira Sampaio Barbosa, Daniele Mendonça Ferreira, Kamilla Carla Bertu Soares, Daniele da Silva Bastos Soares, Patrícia Henriques, Luciene Burlandy

**Affiliations:** I Universidade Federal Fluminense Faculdade de Nutrição Departamento de Nutrição Social NiteróiRJ Brasil Universidade Federal Fluminense. Faculdade de Nutrição. Departamento de Nutrição Social. Niterói, RJ, Brasil; II Universidade Federal Fluminense Faculdade de Nutrição NiteróiRJ Brasil Universidade Federal Fluminense. Faculdade de Nutrição. Curso de graduação em nutrição. Niterói, RJ, Brasil

**Keywords:** Agriculture, Bidding, Food and Nutritional Security, School Feeding, Food and Nutrition Programs and Policies

## Abstract

**OBJECTIVE:**

To analyze how the profile of food purchases from family farming under the National School Feeding Program (PNAE) is related to socioeconomic and demographic indicators in Brazilian capitals.

**METHODS:**

This cross-sectional and descriptive study was based on secondary data from 2016 and 2017 from the Brazilian government. We used demographic and socioeconomic data, as well as the amount of federal funding; the percentage used purchases of food from family farming and the public call notices.

**RESULTS:**

The capitals in the largest quartile of HDI and funding by the federal government used less than 30% of the resource for the purchase of crops from family farming in 2016. All capitals of the Northern region used more than 30%, while the Southern and Southeastern regions did not comply with the legislation. We highlight that most analyzed food items were *in natura*.

**CONCLUSIONS:**

The implementation of this public policy occurs unequally in Brazilian capitals, with greater difficulty in those supposedly with better institutional structure and higher volume of resources destined to the National School Feeding Program. The program, however, maintains its potential for the promotion of adequate and healthy food in schools, due to the quality of food included in public calls.

## INTRODUCTION

The National School Feeding Program (PNAE) is considered an outstanding food and nutrition security policy in Brazil^1^. The recent change in the legal framework of the program included, as a strategy, the mandatory purchase of food from family farms, simultaneously stimulating food production and local sustainability, and expanding the supply of healthy, *in natura* food in schools^2^. Besides, through resolution no. 38/2009^3^, the public call was standardized as a simplified process for the public manager and the farmer, which exempts the bureaucratic chain from bidding ordinarily inaccessible to the segment of family farmers unfamiliar with the bidding requirements process^4^.

The connection of family agriculture with programs that affect access and food quality, such as the PNAE, especially in a context of systematic advancement of obesity, suggests a double potential of this policy design, that is, to improve the quality of school feeding and stimulate the production and local markets of family farming crops. This potential translates into the ability to focus on the perverse consequences of the current food system, characterized by an exclusionary productive model guided by the low diversity and increasing consumption of ultra-processed food items by the population, including schoolchildren exposed to an obesogenic environment^5^.

In Brazil, family agriculture has come to be considered a diverse and heterogeneous social category conceived by government managers and social actors and organizations as strategic in the process of social and economic development. The profile of food produced by the farmers’ segment can be quite diverse, and the demand for market expansion has contributed to the diversification of products with varying degrees of processing^9^. Thus, regarding food processing, food obtained from family-based sources may range from *in natura* crops to food with a high degree of processing and additions of densely caloric, and sugary ingredients.

The enactment of law no. 11,947^2^ increased the farmers’ access to the institutional market through the PNAE. Some studies point to a positive relationship between increase of income and improvement of farmers’ living conditions, diversifying and increasing their production, and improving school meals, with a greater supply of fruits and vegetables^9-11^. Thus, the connection between family-based agriculture and the PNAE enhances changes in the local food system, with possibilities of impact on improving the quality of life of farmers and on the provision of healthy meals for schoolchildren.

However, many challenges are observed when organizing city councils to meet the legal requirements of the program and ensure the supply of healthier food items in schools through the local purchase of family-based crops. We highlight the complexity and diversity of the characteristics of Brazilian cities regarding the structural, political, social, and institutional aspects that can affect the expected potential for the strategy of regulating the public purchase profile.

Brazilian capitals may have advantages and disadvantages concerning less populous and economically less developed municipalities that deserve to be better understood. Besides, the heterogeneity of Brazilian regions and capitals regarding sociodemographic indicators, levels of development, and the number of schoolchildren assisted by the PNAE may represent different challenges and opportunities for the purchase of family-based food still little explored in the literature. Thus, this study aimed to analyze how the food purchase profile of family agriculture is related to socioeconomic and demographic indicators in Brazilian capitals.

## METHODS

This is a cross-sectional and descriptive study, based on secondary data for the years 2016 and 2017 available on the websites of the Brazilian Institute of Geography and Statistics (IBGE)^12^, the National Fund for the Development of Education (FNDE)^13^ and the Ministry of Agrarian Development^14^. Information regarding the capitals’ demographic and socioeconomic profile were: population, territorial area, human development index (HDI), and gross domestic product (GDP), obtained on the IBGE website. We also identified the total of the resource transferred by the FNDE and the percentage used for purchases of food from family farming by Brazilian capitals on the funds’ website. The total value transferred by the FNDE was used as an approximation of the number of students enrolled in the education network since this value is calculated according to the number of enrollments recorded in the year before the transfer. The public call notices were obtained on the ministry’s website, in “Monitoring System of Public Procurement Opportunities of Family Agriculture,” and Transparency Portal of all capitals. Data regarding sociodemographics, FNDE funding, and family agriculture refer to the year 2016, and the purchase notices occurred in 2017.

We also sought to identify the food requested by cafeterias of Brazilian capitals through the analysis of public calls. They were listed and subsequently classified according to the degree of processing, according to the proposed NOVA classification^15^.

In the data analysis, family agriculture purchases were considered as a dependent variable and divided into two categories: percentage of purchases less than 30% and the percentage of purchases greater than or equal to 30%. The Kolmogorov-Smirnov test was applied to identify the normality of distribution of independent continuous variables (HDI, GDP, values transferred from the FNDE, number of inhabitants, and territorial area). The nonparametric variables identified were GDP and number of inhabitants, submitted to the Mann-Whitney test to identify the difference in the median between the categories of family agriculture purchases. For the parametric variables, Student’s t-test was used to identify the difference between the means according to the categories of family agriculture purchases. The parametric variables were expressed in mean and standard deviation and the nonparametric variables in median and quartiles. Variables of FNDE funding, GDP, and HDI, number of inhabitants, and territorial area were organized into distribution quartiles and submitted to the chi-square association test with the categories of purchase of family agriculture. Statistical analysis was performed in the SPSS version 13 program, and, in all tests, the level of significance adopted was 5%.

## RESULTS

### Public Purchases of Family Agriculture for the PNAE in Brazilian Capitals

The average value used in the purchase of family-based crops through the PNAE was higher than that required by the legislation, that is, more than 30% of the money transferred by the FNDE, in 12 Brazilian capitals in 2016. The capitals Boa Vista and Maceió used 100% of the funding, while Rio de Janeiro and Recife did not use any resources with family-based agriculture (Table 1). Only the Northern region of the country presented satisfactory results for the purchase of family-based crops since all capitals met the legal requirements regarding the minimum value destined to this segment. The Southern region has only three capitals, with a smaller territorial area and less allocation of funding for the purchase of family-based crops (Table 1).

**Table 1:** Total funding transferred by the FNDE and percentage used for the purchase of family crops from Brazilian capitals in 2016.

Region	State	Capital	Total funding transferred (R$)	% used with family agriculture
Midwest				
	GO	Goiânia	13,892,920.76	41.10
	MS	Campo Grande	10,232,653.70	13.65
	MT	Cuiabá	6,999,630.59	28.24
	DF	Brasília	44,797,501.27	4.22
North				
	TO	Palmas	10,621,273.97	31.01
	RO	Porto Velho	4,832,239.52	39.02
	AC	Rio Branco	2,887,497.05	35.15
	PA	Belém	6,423,576.23	40.32
	AM	Manaus	22,193,813.59	53.60
	AP	Macapá	2,177,893.98	44.92
	RR	Boa Vista	2,392,953.95	100.00
Northeast				
	PB	João Pessoa	8,697,273.83	10.51
	BA	Salvador	17,015,380.67	1.62
	SE	Aracaju	995,592.74	84.12
	AL	Maceió	170,977.02	100.00
	PE	Recife	8,963,348.14	0.00
	RN	Natal	2,213,052.51	11.02
	CE	Fortaleza	24,438,057.85	9.00
	PI	Teresina	9,416,824.16	46.35
	MA	São Luís	19,808,714.75	27.22
Southeast				
	SP	São Paulo	79,616,147.11	10.75
	RJ	Rio de Janeiro	75,769,080.49	0.00
	ES	Vitória	6,116,291.54	32.62
	MG	Belo Horizonte	20,619,170.14	2.72
South				
	RS	Porto Alegre	9,593,249.88	22.48
	SC	Florianópolis	4,232,436.44	22.57
	PR	Curitiba	21,636,514.96	1.25

The capitals that used the most resources to purchase family crops (≥ 30%) presented lower mean and median values of funding by the FNDE (p = 0.038), HDI (p = 0.021) and number of inhabitants (p = 0.004) than those who used less than 30% of this resource. (Table 2). The analysis of the association between the variables showed that the capitals belonging to the smallest quartiles of income transfer by the FNDE (p = 0.023), HDI (p = 0.005) and number of inhabitants (p = 0.022) are those that buy more family-based crops (> 30%) (Table 3).

**Table 2:** Average and median values of socioeconomic and demographic variables according to the categories of purchase of family crops in 2016.

Variables	% purchases from family agriculture		p
	< 30% (n = 15)	> 30% (n = 12)	
FNDE^a^ funding	207,611,521.8 (20,933,662.9)	6,843,487.9 (6,374,067.9)	0.038
HDI^a^	0.79 (0.30)	0.76 (0.36)	0.021
GDP^b^	34,910.1 (24,029.2; 46,122.8)	24,169.8 (20,520.4; 31,380.0)	0.059
Number of inhabitants^b^	1,633,697.0 (874,210.0; 2,953,986.0)	584,771.0 (368,215.8; 1,346,488.5)	0.004
Territorial area^a^	1,757.4 (2,487.67)	76,792.6 (243,744.1)	0.278

FNDE: National Fund for the Development of Education; HDI: human development index; GDP: gross domestic product

^a^ Parametric variables expressed in mean (standard deviation); Student’s *t-*test.

^b^ Nonparametric variables expressed in median; Mann-Whitney test.

**Table 3:** Distribution of municipalities according to the quartiles of socioeconomic and demographic variables and categories of purchase of family crops in 2016.

Variables	% purchases from family agriculture		p^a^
	< 30% (n = 15)	> 30% (n = 12)	
FNDE Funding			
1º quartil	2 (13.3%)	5 (41.7%)	0.023
2º quartil	3 (20.0%)	4 (33.3%)	
3º quartil	4 (26.7%)	2 (16.7%)	
4º quartil	6 (40.0%)	1 (8.3%)	
HDI			
1º quartil	0 (0%)	6 (50.0%)	0.005
2º quartil	5 (33.3%)	3 (25.0%)	
3º quartil	4 (26.7%)	2 (16.7%)	
4º quartil	6 (40.0%)	1 (8.3%)	
GDP			
1º quartil	2 (13.3%)	5 (41.7%)	0.164
2º quartil	3 (20.0%)	4 (33.3%)	
3º quartil	5 (33.3%)	2 (16.7%)	
4º quartil	5 (33.3%)	1 (8.3%)	
Number of inhabitants			
1º quartil	1 (6.7%)	6 (50.0%)	0.022
2º quartil	4 (26.7%)	3 (25.0%)	
3º quartil	4 (26.7%)	3 (25.0%)	
4º quartil	6 (40.0%)	0 (0%)	
Territorial area			
1º quartil	4 (26.7%)	2 (16.7%)	0.157
2º quartil	6 (40.0%)	2 (16.7%)	
3º quartil	4 (26.7%)	3 (25.0%)	
4º quartil	1 (6.7%)	5 (41.7%)	

FNDE: National Fund for the Development of Education; HDI: human development index; GDP: gross domestic product

^a^ Chi-squared test.

### Public Call Notices and Food Classification

Searches on the ministry’s website and in the Transparency Portal of each of the municipalities allowed the localization of 23 public call notices, totaling 376 items requested for ten Brazilian capitals, during the year 2017. Between those, only four capitals reached the 30% goal of funding spending in family-based agriculture in the previous years, three of them located in the Northern region.

Among the food items required in the notices, 94.1% were classified as *in natura* or minimally processed, 4.0% ultra-processed, and 1.9% processed. The requested crops with a higher degree of processing are primarily those destined for desserts or small meals, such as sweets and flavored dairy products (Table 4).

**Table 4:** Classification of food items requested in public calls from Brazilian capitals in 2017 according to degree of industrial processing.

Degree of processing	Capitals	Number of times food items were requested	Food items
Food *in natura* and minimally processed	Belém	61	Fruits and vegetables, cereals, eggs, pasteurized açaí, starchy goods, tucupi sauce, and others
	Boa Vista	29	Fruits and vegetables, cereals, fruit pulps, small bell peppers, tapioca and honey.
	Campo Grande	34	Fruits, vegetables and cereals
	Fortaleza	8	Fruits, vegetables, cereals and fruit pulps
	João Pessoa	38	Fruits, vegetables, cereals, eggs, fruit pulps and mechanically separated fish meat, among others
	Palmas	20	Fruits, vegetables, cereals and beef
	Rio de Janeiro	97	Fruits, vegetables, cereals and bay leaves
	São Luís	26	Fruits, vegetables, cereals and fruit pulps
	São Paulo	17	Fruits, vegetables, cereals, frozen pork and whole grape juice, among others
	Teresina	24	Fruits, vegetables, cereals and fruit pulps
	Total: 354 (94.1%)		
Processed food items	Fortaleza	1	Curd cheese
	João Pessoa	2	Curd cheese and mozzarella
	Palmas	4	Wheat-based wafers, *cuca* cake, homemade noodles with eggs and homemade bread
	Total: 7 (1.9%)		
Ultra-processed food items	Belém	3	Yogurt and creamy fruit sweets
	Fortaleza	1	Yogurt
	João Pessoa	6	*Doce de leite*, milk-based drinks, light butter and light *requeijão*
	Palmas	3	Pumpkin jam, *doce de leite* and blackberry jam
	São Paulo	2	Milk-based drink, unsalted butter and yogurt
	Total: 15 (4.0%)		

## DISCUSSION

The analysis of the purchasing profile of family-based crops by Brazilian capitals allows us to point out an asymmetry between capitals and regions in compliance with the current legislation of the PNAE and in the potential to stimulate local production and supply of food *in natura* in schools. The analysis of the territorial distribution by regions, considering the differences in terms of area, number of capitals and municipalities by region, points out an absolute heterogeneity.

The Southern region has only three capitals, the smallest territorial area and was the region where the capitals least allocated resources for family farming in 2016. However, the Southern region has a higher percentage of municipalities that meet the minimum criterion of use of the FNDE funding towards family-based agriculture according to different studies^9,10,17,18^. This region has stood out for its rural tradition, which has better organizational and management structures^16^. Studies conducted in municipalities in the three states of the region, Rio Grande do Sul (RS), Santa Catarina (SC) and Paraná (PR), showed that, on average, 70% of the municipalities analyzed in RS and SC used more than 30% of the funding with family farming^17,18^.

The Northern region has seven capitals, including the largest Brazilian capital, Manaus, and was the region that best employed the resource for the purchase of family-based crops. Unequal use of the funding throughout the country seems not to be related to the territorial extension, but possibly to the administrative and management structures that characterize the metropoles. A study conducted in 2012 showed that large municipalities, with mixed, decentralized, or outsourced school feeding management and without a nutritionist as technical responsible, presented a lower frequency of purchase of food from family agriculture^10^.

The characterization of the capitals’ purchasing profile can inform different challenges regarding the institutional systems and processes demanded by metropolitan municipalities that support a higher number of schoolchildren and, therefore, need to mobilize resources on a large scale. It is possible to infer that there is an additional difficulty in purchasing family crops in the capitals compared to the smaller or less populous municipalities. This difference observed in the purchasing profile between the municipalities may suggest that the institutional procedures and the bureaucratic network of the capitals may hinder the articulation between schools, secretariats, and sectors responsible for the fulfillment of the program^19^. The difficulty in allocating resources to family agriculture in more urbanized and developed cities has been highlighted in other studies^16,20^

The specificity of the capital cities may impose difficulties with the processes of purchase of family crops due to the greater distance from agricultural production. Besides, historically, they have more dense and complex bureaucratic management structures, which can delay the adaptation of the new impositions provided for by the PNAE. Capitals mobilize substantial resources in the context of public procurement and, therefore, attract large companies as suppliers. These companies have experience with bidding processes and with political and institutional procedures, which may represent a specific resistance to the entry of new actors in the dispute for access to the public procurement market. The government sectors seem more resistant to change in public procurement mechanisms, which requires new logical and management criteria under the PNAE. In this context, open competition may jeopardize family farms due to institutional relations, companies’ interests and public sectors^5,21^. A recurrent strategy is to use the concept of cost-effectiveness in bid-related legislation^7,22^to justify price definition for family agriculture and thus hinder the participation of these farmers in public calls. It should be noted that the legislation regulating public calls to family agriculture accounts for differentiated pricing criteria and allows the inclusion of the cost of packaging, charges, and logistics in the final price to be paid by the government^7,23^. Nevertheless, misuse of public policy can sometimes cause opportunistic behavior on the part of social agents, either by simulating the condition of a family farmer or, in the case of agrarian cooperatives and associations, appropriation of the farmer’s profit^24^.

Capital cities have better management structures, greater political representativeness for the implementation of new actions, and enormous scope, which seems to be underutilized as a strategy to strengthen family agriculture and the potential supply of healthier food items^25^. Even capitals with extensive experience in the field of food and nutritional security, as is the case of Belo Horizonte, are still far below the legal requirements regarding the use of the FNDE funding^5,21^.

On the other hand, the capitals with the lowest FNDE funding, that is, those with the lowest number of enrolled students can most contribute to the purchase of family crops. They need to manage fewer resources and are sometimes heavily dependent on federal funding.

The capitals grouped in the last quartile of the HDI variable were more associated with the use of less than 30% of the resource with family agriculture; therefore, supposedly more developed capitals are the ones that buy the least crops of this segment. A study conducted in small municipalities in Western Santa Catarina showed that those with a larger territorial area, population, HDI, number of schools, and school enrollments had more difficulty reaching a minimum of 30% in the use of funding^16^. Another study indicates that the oscillation in the municipality’s capacity to comply with the legislation is related to farmers’ production capacity, lack of documentation and the inability to meet the delivery logistics demanded^26^.

It is essential to analyze the type of food primarily demanded in public call notices to understand how the regulation of public purchases impacts the quality of food supply in schools^7^. Family farmers are a heterogeneous group in territorial distribution, management structures, and economic planning over time^5^. Moreover, public call notices are neither homogeneous nor standardized; therefore, although they are designed to facilitate farmer access to the institutional market, depending on how they are drafted and disseminated, they may pose another obstacle for local farmers^27^.

A study conducted in the municipality of Araripe, Ceará, found that the agricultural supply to the PNAE has been predominantly carried out by large companies. It is argued that seasonality, insufficient production volume, and difficulties in logistics due to lack of transportation make it impossible to meet the demands of menus prepared for schools. Thus, most family crops in Araripe that meet the criteria of the public call notices are minimally processed or processed food items, with the addition of sugar and fat. The authors highlight that the rural population of Ceará practices subsistence agriculture and is not able to adapt to the requirements of the PNAE^26^.

The food items prioritized in the analyzed public call notices were classified as *in natura*, and therefore favorable for the provision of a healthier diet in schools. However, other sugary and highly processed food items were also ordered in smaller quantities by five of the capitals, three of those with low funding in family agriculture. It is noteworthy that the legislation of the PNAE does not prohibit the supply of this type of food, although it does limit it^2,23^. Crop perenniality and logistical difficulties, as well as a higher chance of price increase^7^ can sometimes favor the selection of processed or longer shelf-time food items, such as sweets, to ensure compliance with the legislation, since financial penalties are expected for states and municipalities that do not meet legal requirements without justification^28^. However, the manager should consider the existence of public agencies’ specific health legislation for the purchase of processed or ultra-processed food items^23^.

The PNAE, although very promising and with significant advances, still only represents an alternative market for the family farmer ^29,30^. The institutionalization of the purchase of food *in natura* primarily through the supply of family-based farmers needs to be signified in the context of the public management of financial resources allocated to the PNAE by the FNDE, and the agencies responsible for public purchases must understand the purposes and principles that guide law no. 11,947/2009^2^, especially in Brazilian capitals.

Metropoles such as capitals have specificities that require additional investment in infrastructure to meet the logistics demand and intersectoral articulation strategies that involve the sectors responsible for public procurement, policy managers, nutritionists, and farmers, as well as technical assistance agencies focused on rural extension throughout the process. The success and full development of this public policy can impact various social benefits, either by strengthening local food production and markets from family-based farmers or by providing fresher and healthier food for schoolchildren. The qualification of this process may represent the possibility of reorienting the logic of the sectors responsible for public purchases towards new principles that go outside the economic perspective in favor of valuing social gains.

## CONCLUSION

The purchase of family crops for the PNAE has advanced in the country; however, it still occurs unevenly in Brazilian capitals, and the resource is used irregularly and unsatisfactory in most regions. Compliance with the minimum criteria established in the legislation on the use of resources for family agriculture is inversely related to metropolitan municipalities’ socioeconomic and demographic indicators.

The number of public calls available to access is small if the total resources transferred to the capitals are considered and are therefore insufficient to meet the supply demands of schools and pretensions regarding the inclusion of family farmers in the PNAE. It is noteworthy that the disclosure of public calls is still limited, even in municipalities with more considerable institutional and financial resources. Most food items *in natura* or minimally processed may represent the potential for the promotion of adequate and healthy food in schools provided for the PNAE, strengthening it as an essential strategy to promote health in the school context. We highlight the limitations of a study based on secondary data, which, although it offers a national overview of how socioeconomic and demographic indicators related to the execution of institutional purchases of PA for the PNAE, lacks analyses on the specificities and institutional characteristics that may facilitate or hinder compliance with the legislation in force in the capitals of the country. Therefore, an influential research agenda in this area of public policies is suggested.
