# Supplementary material for: Purchases from family agriculture for school feeding in Brazilian capitals
Source: Rev Saude Publica. 2020 Jul 20;54:73. doi: 10.11606/s1518-8787.2020054001963 (PMC7371408; doi:10.11606/s1518-8787.2020054001963)
Supplement: Translation

# Translation: Compra da agricultura familiar para alimentação escolar nas capitais brasileiras

**Authors:** Patricia Camacho Dias, Isis Ribeiro de Oliveira Barbosa, Roseane Moreira Sampaio Barbosa, Daniele Mendonça Ferreira, Kamilla Carla Bertu Soares, Daniele da Silva Bastos Soares, Patrícia Henriques, Luciene Burlandy

**Affiliations:** I Brasil Universidade Federal Fluminense. Faculdade de Nutrição. Departamento de Nutrição Social. Niterói, RJ, Brasil; II Brasil Universidade Federal Fluminense. Faculdade de Nutrição. Curso de graduação em nutrição. Niterói, RJ, Brasil

**Keywords:** Agricultura, Licitação, Segurança Alimentar e Nutricional, Alimentação Escolar, Programas e Políticas de Nutrição e Alimentação

## Abstract

**OBJETIVO:**

Analisar como o perfil de compra de alimentos da agricultura familiar no âmbito do Programa Nacional de Alimentação Escolar se relaciona com indicadores socioeconômicos e demográficos nas capitais brasileiras.

**MÉTODOS:**

Este estudo transversal e descritivo foi baseado em dados secundários de 2016 e 2017 do governo brasileiro. Foram utilizados dados demográficos e socioeconômicos, o valor do recurso repassado pelo governo federal, o percentual utilizado para compras de alimentos da agricultura familiar e as chamadas públicas disponíveis.

**RESULTADOS:**

As capitais no maior quartil de índice de desenvolvimento humano e de recursos repassados pelo governo federal utilizaram menos de 30% do recurso para a compra de gêneros da agricultura familiar em 2016. Todas as capitais da região Norte utilizaram acima de 30%, enquanto as regiões Sul e Sudeste não atenderam à legislação. Destaca-se a presença majoritária de alimentos *in natura* nas chamadas públicas analisadas.

**CONCLUSÕES:**

A execução dessa política pública ocorre de forma desigual nas capitais brasileiras, com maior dificuldade naquelas supostamente com melhor estrutura institucional e maior volume de recursos destinados ao Programa Nacional de Alimentação Escolar, contudo, o programa mantém seu potencial para a promoção da alimentação adequada e saudável nas escolas, em razão da qualidade dos alimentos incluídos nas chamadas públicas.

## INTRODUÇÃO

O Programa Nacional de Alimentação Escolar (PNAE) é considerado uma importante política de segurança alimentar e nutricional no Brasil^1^. A recente mudança no marco legal do programa incluiu, como estratégia, a obrigatoriedade de compra de alimentos da agricultura familiar (AF), com o propósito de estimular simultaneamente a produção de alimentos e a sustentabilidade local, assim como ampliar a oferta de alimentos *in natura* e saudáveis nas escolas^2^. Além disso, por meio da resolução nº 38/2009^3^, normatizou-se a chamada pública (CP) como um processo simplificado para o gestor público e para o agricultor, que dispensa a cadeia burocrática da licitação normalmente inacessível para o segmento de agricultores familiares não familiarizados com as exigências do processo licitatório^4^.

A conexão da AF com programas que afetam a dimensão do acesso e a qualidade de alimentos, como o PNAE, sobretudo em um contexto de avanço sistemático da obesidade, sugere um duplo potencial desse desenho de política, qual seja, melhorar a qualidade da alimentação escolar e estimular a produção e mercados locais de gêneros da AF. Tal potencial se traduz na capacidade de incidir sobre as consequências perversas do sistema alimentar vigente, caracterizado por um modelo produtivo excludente orientado pela pouca diversidade e pelo consumo crescente de alimentos ultraprocessados pela população, inclusive por escolares (crianças) expostos a um ambiente obesogênico^5^.

No Brasil, AF passou a ser considerada uma categoria social diversa e heterogênea concebida pelos gestores governamentais e pelos atores e organizações sociais como estratégica no processo de desenvolvimento social e econômico. O perfil de alimentos produzidos pelo segmento de agricultores pode ser bastante diverso, e a demanda de ampliação de mercado tem contribuído para a diversificação de produtos com graus variados de beneficiamento^9^. Assim, considerando o processamento dos alimentos, é possível encontrar desde alimentos *in natura* até alimentos com elevado grau de processamento ou adição de ingredientes densamente calóricos e açucarados no rol de produtos da AF.

A promulgação da lei nº 11.947^2^ ampliou o acesso dos agricultores ao mercado institucional por meio do PNAE. Alguns estudos apontam uma relação positiva entre o aumento de renda e a melhoria das condições de vida dos agricultores, diversificação e aumento da sua produção e melhoria da alimentação escolar, com maior oferta de frutas, legumes e verduras^9^. Desse modo, a conexão entre agricultura de base familiar e o PNAE potencializa as mudanças no sistema alimentar local, com possibilidades de impacto na melhoria da qualidade de vida de agricultores e na oferta de refeições saudáveis para os escolares.

No entanto, muitos desafios são observados no processo de organização dos municípios para atender às exigências legais do programa e garantir a oferta de alimentos mais saudáveis nas escolas por meio da compra local da AF. Destacam-se a complexidade e a diversidade das características dos municípios brasileiros quanto aos aspectos estruturais, políticos, sociais e institucionais que podem afetar o potencial esperado para a estratégia de regulação do perfil de compras públicas.

Considera-se que as capitais brasileiras podem assumir vantagens e desvantagens em relação aos municípios menos populosos e economicamente menos desenvolvidos que merecem ser melhor compreendidas. Além disso, a heterogeneidade das regiões e capitais brasileiras quanto aos indicadores sociodemográficos, níveis de desenvolvimento e o quantitativo de escolares atendidos pelo PNAE podem representar diferentes desafios e oportunidades para a compra de alimentos da AF ainda pouco explorados na literatura. Assim, o objetivo deste estudo foi analisar como o perfil de compra de alimentos da AF se relaciona com indicadores socioeconômicos e demográficos nas capitais brasileiras.

## MÉTODOS

Trata-se de um estudo transversal e descritivo, baseado em dados secundários referentes aos anos de 2016 e 2017 disponíveis nos sítios eletrônicos do Instituto Brasileiro de Geografia e Estatística (IBGE)^12^, do Fundo Nacional de Desenvolvimento da Educação (FNDE)^13^ e do Ministério do Desenvolvimento Agrário (MDA)^14^. As informações referentes ao perfil demográfico e socioeconômico das capitais foram: população, área territorial, índice de desenvolvimento humano (IDH) e produto interno bruto (PIB), obtidas no sítio eletrônico do IBGE. Identificaram-se também o total do recurso repassado pelo FNDE e o percentual utilizado para compras de alimentos provenientes da agricultura familiar pelas capitais brasileiras no sítio eletrônico do fundo. O valor total do recurso transferido pelo FNDE foi utilizado como uma aproximação do quantitativo de alunos matriculados na rede de ensino, uma vez que este valor é calculado em função da quantidade de matrículas registradas no ano anterior ao repasse. Os editais de CP foram obtidos no sítio eletrônico do MDA, na seção “Sistema de Monitoramento de Oportunidades de Compras Públicas da Agricultura Familiar”, e no Portal da Transparência de todas as capitais. Os dados sociodemográficos e os dados dos valores de verbas do FNDE e AF são referentes ao ano de 2016 e os dados de editais de compra são referentes ao ano de 2017.

Buscou-se ainda identificar, por meio da análise das chamadas públicas os alimentos solicitados para a alimentação escolar pelas capitais brasileiras. Eles foram listados e posteriormente classificados em função do grau de processamento, conforme proposta de classificação NOVA ^15^.

Na análise dos dados, a compra da AF foi considerada como variável dependente e dividida em duas categorias: percentual de compras menor que 30% e percentual de compras maior ou igual a 30%. Para identificar a normalidade de distribuição das variáveis contínuas independentes (IDH, PIB, valores transferidos do FNDE, número de habitantes e área territorial) foi aplicado o teste de Kolmogorov-Smirnov. As variáveis não paramétricas identificadas foram PIB e número de habitantes, submetidas ao teste de Mann-Whitney para identificação da diferença da mediana entre as categorias de compra da AF. Para as variáveis paramétricas, utilizou-se o teste *t* de Student para identificação da diferença entre as médias segundo categorias de compra da AF. As variáveis paramétricas foram expressas em média e desvio-padrão, e as variáveis não paramétricas em mediana e quartis. Os valores transferidos do FNDE, valores de IDH e de PIB, número de habitantes e área territorial foram organizados em quartis de distribuição e submetidos ao teste de associação qui-quadrado com as categorias de compra da AF. A análise estatística foi realizada no programa SPSS versão 13 e, em todos os testes, o nível de significância adotado foi de 5%.

## RESULTADOS

### Compras Públicas da Agricultura Familiar para o PNAE nas Capitais Brasileiras

O valor médio utilizado na compra de gêneros da AF por meio do PNAE foi superior ao exigido pela legislação, isto é, superior a 30% da verba repassada pelo FNDE, em 12 capitais brasileiras no ano de 2016. As capitais Boa Vista e Maceió utilizaram 100% do recurso repassado pelo FNDE, enquanto Rio de Janeiro e Recife não utilizaram nenhum recurso com a AF (Tabela 1). Apenas a região Norte do país apresentou resultado satisfatório para a compra de gêneros da AF, uma vez que todas as capitais cumpriram as exigências legais quanto ao valor mínimo destinado a esse segmento. A região Sul possui apenas três capitais, que apresentam menor área territorial e menor destinação dos recursos para a AF (Tabela 1).

**Tabela 1:** Total do recurso repassado pelo Fundo Nacional de Desenvolvimento da Educação e percentual utilizado para a compra de gêneros da agricultura familiar (AF) das capitais brasileiras no ano de 2016.

Região	Estado	Capital	Total do recurso repassado (R$)	% utilizado com a AF
Centro-Oeste				
	GO	Goiânia	13.892.920,76	41,10
	MS	Campo Grande	10.232.653,70	13,65
	MT	Cuiabá	6.999.630,59	28,24
	DF	Brasília	44.797.501,27	4,22
Norte				
	TO	Palmas	10.621.273,97	31,01
	RO	Porto Velho	4.832.239,52	39,02
	AC	Rio Branco	2.887.497,05	35,15
	PA	Belém	6.423.576,23	40,32
	AM	Manaus	22.193.813,59	53,60
	AP	Macapá	2.177.893,98	44,92
	RR	Boa Vista	2.392.953,95	100,00
Nordeste				
	PB	João Pessoa	8.697.273,83	10,51
	BA	Salvador	17.015.380,67	1,62
	SE	Aracaju	995.592,74	84,12
	AL	Maceió	170.977,02	100,00
	PE	Recife	8.963.348.14	0,00
	RN	Natal	2.213.052,51	11,02
	CE	Fortaleza	24.438.057,85	9,00
	PI	Teresina	9.416.824,16	46,35
	MA	São Luís	19.808.714,75	27,22
Sudeste				
	SP	São Paulo	79.616.147,11	10,75
	RJ	Rio de Janeiro	75.769.080,49	0,00
	ES	Vitória	6.116.291,54	32,62
	MG	Belo Horizonte	20.619.170,14	2,72
Sul				
	RS	Porto Alegre	9.593.249,88	22,48
	SC	Florianópolis	4.232.436,44	22,57
	PR	Curitiba	21.636.514,96	1,25

As capitais que mais utilizaram recursos para compra da AF (≥ 30%) apresentaram menores valores médios e medianos de transferência de recurso pelo FNDE (p = 0,038), IDH (p = 0,021) e número de habitantes (p = 0,004) do que aquelas que utilizaram menos que 30% desse recurso para compra de gêneros da AF (Tabela 2). A análise da associação entre as variáveis mostrou que as capitais pertencentes aos menores quartis de transferência de recurso pelo FNDE (p = 0,023), IDH (p = 0,005) e número de habitantes (p = 0,022) são aquelas que mais compram gêneros da AF (> 30%) (Tabela 3).

**Tabela 2:** Valores médios e medianos das variáveis socioeconômicas e demográficas de acordo com as categorias de compra de gêneros da agricultura familiar (AF) no ano de 2016.

Variáveis	% compras da AF		p
	< 30% (n = 15)	> 30% (n = 12)	
Transferência do FNDE^a^	207.611.521,8 (20.933.662,9)	6.843.487,9 (6.374.067,9)	0,038
IDH^a^	0,79 (0,30)	0,76 (0,36)	0,021
PIB^b^	34.910,1 (24.029,2; 46.122,8)	24.169,8 (20.520,4; 31.380,0)	0,059
Número de habitantes^b^	1.633.697,0 (874.210,0; 2.953.986,0)	584.771,0 (368.215,8; 1.346.488,5)	0,004
Área territorial^a^	1.757,4 (2.487,67)	76.792,6 (243.744,1)	0,278

FNDE: Fundo Nacional de Desenvolvimento da Educação; IDH: índice de desenvolvimento humano; PIB: produto interno bruto

^a^ Variáveis paramétricas expressas em média (desvio-padrão); teste *t* de Student.

^b^ Variáveis não paramétricas expressas em mediana (p25; p75); teste de Mann-Whitney.

**Tabela 3:** Distribuição dos municípios de acordo com os quartis das variáveis socioeconômicas e demográficas e categorias de compra de gêneros da agricultura familiar (AF) no ano de 2016.

Variáveis	% compras da AF		p*
	< 30% (n = 15)	> 30% (n = 12)	
Transferência do FNDE			
1º quartil	2 (13,3%)	5 (41,7%)	0,023
2º quartil	3 (20,0%)	4 (33,3%)	
3º quartil	4 (26,7%)	2 (16,7%)	
4º quartil	6 (40,0%)	1 (8,3%)	
IDH			
1º quartil	0 (0%)	6 (50,0%)	0,005
2º quartil	5 (33,3%)	3 (25,0%)	
3º quartil	4 (26,7%)	2 (16,7%)	
4º quartil	6 (40,0%)	1 (8,3%)	
PIB			
1º quartil	2 (13,3%)	5 (41,7%)	0,164
2º quartil	3 (20,0%)	4 (33,3%)	
3º quartil	5 (33,3%)	2 (16,7%)	
4º quartil	5 (33,3%)	1 (8,3%)	
Número de habitantes			
1º quartil	1 (6,7%)	6 (50,0%)	0,022
2º quartil	4 (26,7%)	3 (25,0%)	
3º quartil	4 (26,7%)	3 (25,0%)	
4º quartil	6 (40,0%)	0 (0%)	
Área territorial			
1º quartil	4 (26,7%)	2 (16,7%)	0,157
2º quartil	6 (40,0%)	2 (16,7%)	
3º quartil	4 (26,7%)	3 (25,0%)	
4º quartil	1 (6,7%)	5 (41,7%)	

FNDE: Fundo Nacional de Desenvolvimento da Educação; IDH: índice de desenvolvimento humano; PIB: produto interno bruto

* Teste qui-quadrado.

### Chamadas Públicas e a Classificação de Alimentos

A busca no sítio do MDA e no Portal da Transparência de cada um dos municípios permitiu localizar 23 CP, totalizando 376 itens solicitados para dez capitais brasileiras, referentes ao ano de 2017. Dessas, apenas quatro atingiram os 30% da utilização do recurso do FNDE com gêneros da AF no ano anterior, sendo três delas localizadas na região Norte do país.

Dentre os alimentos requeridos nos editais, 94,1% foram classificados como *in natura* ou minimamente processados, 4,0% ultraprocessados e 1,9% processados. Os gêneros solicitados com maior grau de processamento são prioritariamente aqueles destinados às sobremesas ou pequenas refeições, tais como doces e derivados de leite acrescidos de sabor (Tabela 4).

**Tabela 4:** Classificação do grau de processamento segundo a NOVA15 dos gêneros alimentícios solicitados nas chamadas públicas das capitais do Brasil no ano de 2017.

Grau de processamento	Capitais	Nº de vezes que os gêneros alimentícios foram solicitados	Gêneros alimentícios
Alimentos *in natura* e minimamente processados	Belém	61	Frutas, hortaliças, leguminosas, cereais, ovo, açaí pasteurizado, farináceos e tucupi, entre outros
	Boa Vista	29	Frutas, hortaliças, leguminosas, cereais, polpa de fruta, pimenta-de-cheiro, goma de tapioca e mel de abelha
	Campo Grande	34	Frutas, hortaliças, leguminosas e cereais
	Fortaleza	8	Frutas, hortaliças, leguminosas, cereais e polpa de fruta
	João Pessoa	38	Frutas, hortaliças, leguminosas, cereais, ovo, polpa de fruta e carne de peixe mecanicamente separada, entre outros
	Palmas	20	Frutas, hortaliças, leguminosas cereais e carne bovina
	Rio de Janeiro	97	Frutas, hortaliças, leguminosas, cereais e louro
	São Luís	26	Frutas, hortaliças, leguminosas, cereais e polpa de fruta
	São Paulo	17	Frutas, hortaliças, leguminosas, cereais, carne suína congelada e suco de uva integral, entre outros
	Teresina	24	Frutas, hortaliças, leguminosas, cereais e polpa de fruta
	Total: 354 (94,1%)		
Alimentos processados	Fortaleza	1	Queijo coalho
	João Pessoa	2	Queijo coalho e queijo muçarela
	Palmas	4	Bolacha doce de trigo, cuca, macarrão caseiro com ovos e pão caseiro
	Total: 7 (1,9%)		
Alimentos ultraprocessados	Belém	3	Iogurte e doce de fruta cremoso
	Fortaleza	1	Iogurte
	João Pessoa	6	Doce de leite, bebida láctea, manteiga light e requeijão light
	Palmas	3	Doce de abóbora, doce de leite e geleia de amora
	São Paulo	2	Bebida láctea, manteiga sem sal e iogurte
	Total: 15 (4,0%)		

## DISCUSSÃO

A análise do perfil de compra de gêneros da AF pelas capitais brasileiras permite apontar uma assimetria entre as capitais e regiões no cumprimento da legislação vigente do PNAE e no potencial de estímulo à produção local e oferta de alimentos *in natura* nas escolas. A análise da distribuição territorial por regiões, considerando as diferenças em termos de área, número de capitais e municípios por região, aponta certa heterogeneidade.

A região Sul possui apenas três capitais, constitui a menor área territorial e foi a região em que as capitais menos destinaram recursos para a agricultura familiar em 2016. No entanto, o Sul apresenta maior percentual de municípios que atendem ao critério mínimo de utilização do recurso do FNDE com a AF segundo diferentes estudos^9,10,17,18^. Essa região tem se destacado por sua tradição rural, que possui melhores níveis de organização e estruturas de gestão^16^. Estudos realizados em municípios dos três estados da região, Rio Grande do Sul (RS), Santa Catarina (SC) e Paraná (PR), mostraram que, em média, 70% dos municípios analisados no RS e em SC utilizaram mais de 30% do recurso com a AF^17,18^.

A região Norte possui sete capitais, incluindo a maior capital brasileira, Manaus, e foi a região que melhor empregou o recurso para a compra de gêneros da agricultura familiar. O emprego desigual do recurso nessa compra em todo o território nacional parece não estar relacionado com a extensão territorial, mas possivelmente com as estruturas administrativas e de gestão que caracterizam as metrópoles. O resultado de um estudo realizado em 2012 apontou que municípios de grande porte, com gestão da alimentação escolar do tipo mista, descentralizada ou terceirizada e sem nutricionista como responsável técnico, apresentaram menor frequência de compra de alimentos da agricultura familiar^10^.

A caracterização do perfil de compra pelas capitais pode informar diferentes desafios quanto aos sistemas e processos institucionais demandados por municípios metropolitanos que atendem a um quantitativo elevado de escolares e, portanto, mobilizam recursos em larga escala. É possível inferir que existe uma dificuldade adicional para o processo de compra de alimentos da AF nas capitais em comparação com os municípios menores e ou menos populosos. Essa diferença observada no perfil de compra entre os municípios pode sugerir que os trâmites institucionais e a rede burocrática das capitais podem dificultar a articulação entre escolas, secretarias e setores responsáveis para o cumprimento do programa^19^. A dificuldade na destinação de recursos para a AF em cidades mais urbanizadas e desenvolvidas vem sendo destacada em outros estudos^16,20^.

A especificidade das capitais pode impor dificuldades com os processos de compra da AF em razão da maior distância da produção agrícola. Além disso, historicamente, possuem estruturas burocráticas de gestão mais densas e complexas, que podem retardar a adaptação das novas imposições previstas pelo PNAE. As capitais mobilizam recursos vultuosos no âmbito das compras públicas e, portanto, atraem empresas de grande porte como fornecedores. Tais empresas possuem experiência com processos licitatórios e com os trâmites políticos e institucionais, o que pode representar certa resistência à entrada de novos atores na disputa por acesso ao mercado das compras públicas. Os setores de governo parecem mais refratários a uma mudança nos mecanismos de compras públicas, que exige novas lógicas e critérios de gestão no âmbito do PNAE. A abertura de concorrência para a AF nesse contexto pode ser dificultada pelas relações institucionais, os interesses das empresas e dos setores públicos^5,21^. Nesse sentido, é recorrente o uso do princípio da economicidade que rege a legislação que trata de licitação^7,22^ para justificar a definição de preços para AF e assim dificultar a participação dos agricultores nas CP. Cabe destacar que a legislação que regulamenta as chamadas públicas para AF prevê critérios de definição de preços diferenciados e possibilita ainda a inclusão do custo com embalagens, encargos e logística no preço final a ser pago pelo setor público^7,23^. Não obstante, o uso indevido da política pública pode ocasionar por vezes comportamento oportunista por parte de agentes sociais, seja simulando a condição de agricultor familiar ou, no caso de cooperativas e associações, a apropriação do lucro do agricultor^24^.

As capitais possuem melhores estruturas de gestão, maior representatividade política para a implementação de novas ações e maior abrangência, que parece estar sendo subaproveitada como estratégia de fortalecimento da AF e de potencial oferta de alimentos mais saudáveis^25^. Mesmo aquelas capitais com larga experiência no campo da segurança alimentar e nutricional, como é o caso de Belo Horizonte, ainda se encontram muito aquém das exigências legais quanto à utilização do recurso do FNDE^5,21^.

Já as capitais com menores valores de repasse do FNDE, ou seja, aquelas com menor quantitativo de alunos matriculados, são as que mais conseguem aportar recursos na compra de gêneros da AF. Elas precisam gerir menor volume de recursos e por vezes são fortemente dependentes de recursos federais.

As capitais agrupadas no último quartil da variável IDH foram mais associadas à utilização de menos do que 30% do recurso com a AF; portanto, capitais supostamente mais desenvolvidas são as que menos compram gêneros desse segmento. Em um estudo realizado em municípios de pequeno porte da mesorregião Oeste Catarinense apontou que aqueles com maior área territorial, população, IDH, número de escolas e matrículas escolares tiveram mais dificuldade em atingir o mínimo de 30%^16^. Outro estudo aponta que a oscilação na capacidade de o município atender a legislação está relacionada com a capacidade de produção dos agricultores, falta de documentação e incapacidade para atender à logística de entrega demandada^26^.

É importante analisar o tipo de alimento prioritariamente demandado nas CP para compreender como a regulação das compras públicas impacta na qualidade da oferta de alimentos nas escolas^7^. A categoria de agricultores familiares é bastante heterogênea e se diferencia em distribuição territorial, estruturas de gestão e planejamento econômico ao longo do tempo^5^. Além disso, os instrumentos de CP também não são homogêneos e nem padronizados; portanto, embora sejam concebidos para facilitar o acesso do agricultor ao mercado institucional, dependendo de como são redigidos e divulgados, podem representar mais um obstáculo para os agricultores locais^27^.

Um estudo realizado no município de Araripe, no Ceará, constatou que o fornecimento agrícola para o PNAE vem sendo predominantemente realizado por grandes empresas. Argumenta-se que a sazonalidade, o volume insuficiente da produção e as dificuldades na logística pela falta de transporte inviabilizam o atendimento das demandas dos cardápios elaborados para as escolas. Assim, a maioria dos gêneros oriundos da AF em Araripe que conseguem cumprir os critérios dos editais de CP são alimentos minimamente processados ou processados, com adição de açúcar e gordura. Os autores destacam que a população rural da região do Ceará pratica a agricultura de subsistência, não sendo ainda capaz de adequar-se às exigências do PNAE^26^.

Os alimentos priorizados nas CP analisadas foram classificados como *in natura*, sendo, portanto, favoráveis para a oferta de uma alimentação mais saudável nas escolas. Contudo, alguns outros alimentos ricos em açúcar e alguns processados e ultraprocessados também foram solicitados em menor quantidade por cinco capitais, das quais três com baixo investimento do recurso na AF. Destaca-se que a legislação do PNAE, ainda que limite, não proíbe a oferta desse tipo de alimento^2,23^. A perenidade das frutas e hortaliças associada às dificuldades na logística de entrega e maior possibilidade de agregação de valor comercial^7^ por vezes pode favorecer a inclusão de alimentos processados ou com maior tempo de prateleira, como doces, para garantir o cumprimento do uso dos 30% dos recursos previstos na legislação, uma vez que existe a previsão de penalidades financeiras para os estados e municípios que não atendem às exigências legais sem justificativa^28^. No entanto, o gestor deve considerar a existência de legislações sanitárias específicas dos órgãos públicos para a compra de alimentos processados ou ultraprocessados^23^.

O PNAE, embora muito promissor e com significativos avanços, ainda representa um canal de comercialização apenas alternativo para o agricultor familiar^29,30^. A institucionalização da compra de alimentos *in natura* prioritariamente por meio da oferta dos agricultores de base familiar precisa ser significada no âmbito da gestão pública dos recursos financeiros destinados ao PNAE pelo FNDE, sendo fundamental que os órgãos responsáveis pelas compras públicas compreendam os propósitos e princípios que orientam a lei nº 11.947/2009^2^, especialmente nas capitais brasileiras.

Os municípios de regiões metropolitanas, como é o caso das capitais, possuem especificidades e necessitam de investimento adicional em infraestrutura para atender à demanda logística e em estratégias de articulação intersetorial que envolva os setores responsáveis pelas compras públicas, gestores de política, nutricionistas e agricultores, bem como os órgãos de assistência técnica voltados para a extensão rural em todo o processo. O sucesso e o pleno desenvolvimento dessa política pública podem impactar em vários benefícios sociais, seja pelo fortalecimento da produção e dos mercados locais de alimentos provenientes de agricultores de base familiar, seja pela oferta de alimentos mais frescos e saudáveis para escolares. A qualificação desse processo pode representar a possibilidade de reorientar a lógica dos setores responsáveis pelas compras públicas em direção a novos princípios que extrapolem a perspectiva econômica em favor da valorização dos ganhos sociais.

## CONCLUSÃO

A compra de gêneros da AF para o PNAE avançou no país; contudo, nas capitais brasileiras ainda ocorre de forma desigual, e o recurso é empregado de forma irregular e insatisfatória na maioria das regiões. O cumprimento dos critérios mínimos estabelecidos na legislação do programa acerca da utilização dos recursos para AF está inversamente relacionado aos indicadores socioeconômicos e demográficos dos municípios metropolitanos.

O número de CP disponíveis ao acesso público é pequeno se considerado o total dos recursos repassados para as capitais, sendo, portanto, insuficiente para atender às demandas de abastecimento das escolas e as pretensões quanto à inclusão dos agricultores familiares no PNAE. Destaca-se que divulgação das CP ainda é limitada, mesmo nos municípios com maiores recursos institucionais e financeiros. A presença majoritária de alimentos *in natura* ou minimamente processados pode representar o alcance do potencial para a promoção da alimentação adequada e saudável nas escolas previsto para o PNAE, fortalecendo-o como uma importante estratégia de promoção da saúde no contexto escolar. Destacam-se as limitações de um estudo baseado em dados secundários, que, ainda que ofereça um panorama nacional de como os indicadores socioeconômicos e demográficos se relacionam com a execução das compras institucionais da AF para o PNAE, carece de análises sobre as especificidades e características institucionais que podem facilitar ou dificultar o cumprimento da legislação vigente nas capitais do país. Sugere-se, portanto, uma importante agenda de pesquisa nessa área de políticas públicas.
